# The Infrapopliteal Arterial Occlusions Similar to Buerger Disease: Report of Two Cases

**DOI:** 10.1155/2014/874528

**Published:** 2014-11-06

**Authors:** Kimihiro Igari, Toshifumi Kudo, Takahiro Toyofuku, Yoshinori Inoue, Takehisa Iwai

**Affiliations:** ^1^Division of Vascular and Endovascular Surgery, Department of Surgery, Tokyo Medical and Dental University, 1-5-45 Yushima, Bunkyo-ku, Tokyo 113-8519, Japan; ^2^Tsukuba Vascular Center and Buerger Disease Research Institute, 980-1 Tatsuzawa, Moriya, Ibaraki 302-0118, Japan

## Abstract

We herein present two cases that required the differential diagnosis of Buerger disease. Case 1 involved a 55-year-old male with a smoking habit who was admitted with ulcers and coldness in his fingers and toes. Angiography showed blockage in both the radial and posterior tibial arteries, which led to an initial diagnosis of Buerger disease. However, a biopsy of the right posterior tibial artery showed pathological findings of fibromuscular dysplasia (FMD). Case 2 involved a 28-year-old male with intermittent claudication who was examined at another hospital. Angiography showed occlusion of both popliteal and crural arteries, and the patient was suspected to have Buerger disease. However, computed tomography disclosed an abnormal slip on both sides of the popliteal fossa, and we diagnosed him with bilateral popliteal artery entrapment syndrome (PAES). These cases illustrate that other occlusive diseases, such as FMD and PAES, may sometimes be misdiagnosed as Buerger disease.

## 1. Introduction

Buerger disease is a nonatherosclerotic inflammatory occlusive disease [1], which most commonly affects the small- and medium-sized arteries and veins of the upper and lower extremities [2]. Most of patients with Buerger disease are young males, typically <50 years of age [3], and are usually heavy cigarette smokers, with no atherosclerotic risk factors other than smoking.

Buerger disease is one of the many diseases presenting with ischemic symptoms that manifest as intermittent claudication, rest pain, ulceration, and/or gangrene. Since the specific clinical features of Buerger disease are characterized by peripheral ischemia, the diagnostic criteria should be discussed from the clinical point of view. However, the clinical criteria used to diagnose Buerger disease remain controversial [4], and some authors have stated that the diagnosis of Buerger disease requires arteriography and biopsy assessments of the affected arteries [5], which makes it difficult to diagnose Buerger disease precisely. We herein present two cases of lower extremity ischemic symptoms that required a differential diagnosis of Buerger disease.

## 2. Case Presentation

**Figure 1:**
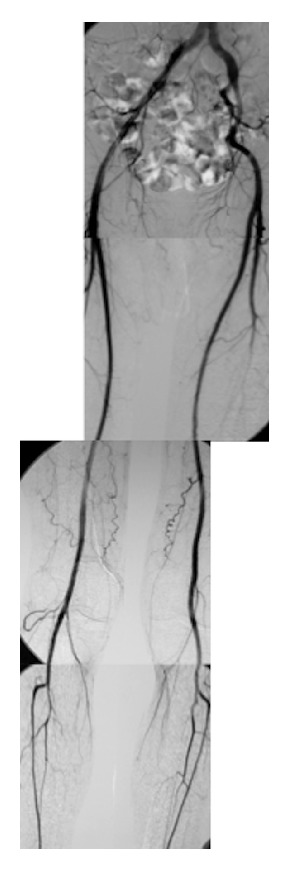
Case 1: preoperative arteriography showed that the bilateral iliac, femoral, and popliteal arteries were intact, but the bilateral posterior tibial arteries were occluded.

*Case 1.* A 55-year-old male with a smoking habit was admitted with ulcers and coldness in his fingers and toes. His medical history was unremarkable, without diabetes mellitus, hyperlipidemia, or hypertension. During the clinical examination, his third and fourth fingers on the left side were observed to be ulcerated, and his fourth right toe was gangrenous. The bilateral radial and posterior tibial arteries were not palpable; however, the ankle brachial index (ABI) was within the normal range on both sides. The laboratory findings failed to show any thrombophilia, autoimmune disorders, or malignant diseases. However, arteriography disclosed blockage in both the radial and posterior tibial arteries, which led to an initial diagnosis of Buerger disease (Figure 1).

With respect to the Shionoya diagnostic criteria [6], the patient had a history of smoking, and the initial onset of symptoms occurred at 49 years of age. The infrapopliteal and upper extremity arteries were affected, and he had no risk factors for atherosclerosis, except for his smoking habit. Therefore, we diagnosed him as having Buerger disease.

**Figure 2:**
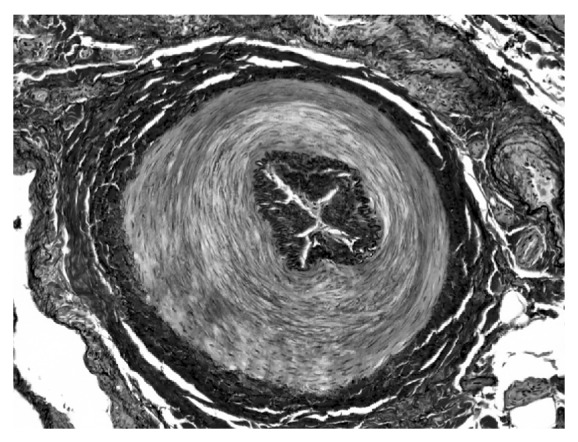
A biopsy specimen of the right posterior tibial artery in Case 1. The arterial wall in the media was thickened. There was no evidence of infiltration of inflammatory cells (Elastica van Gieson stain, ×40).

We performed sympathectomy of the left thoracic sympathetic nerve and the right lumbar sympathetic nerve, with a simultaneous biopsy of the right posterior tibial artery. The patient's postoperative course was uneventful, and the ulceration and gangrene healed successfully. On a histopathological examination of the right posterior tibial artery, no organized clotting or venous thrombophlebitis were observed. The resected arterial wall was thickened in the media and hyperplastic in the adventitia and there were no inflammatory cells (Figure 2). These findings suggested that the patient had fibromuscular dysplasia (FMD), rather than Buerger disease. As a result, he was initially diagnosed with Buerger disease based on the Shionoya diagnostic criteria and then finally diagnosed with FMD based on the pathological findings of the peripheral arteries.

**Figure 3:**
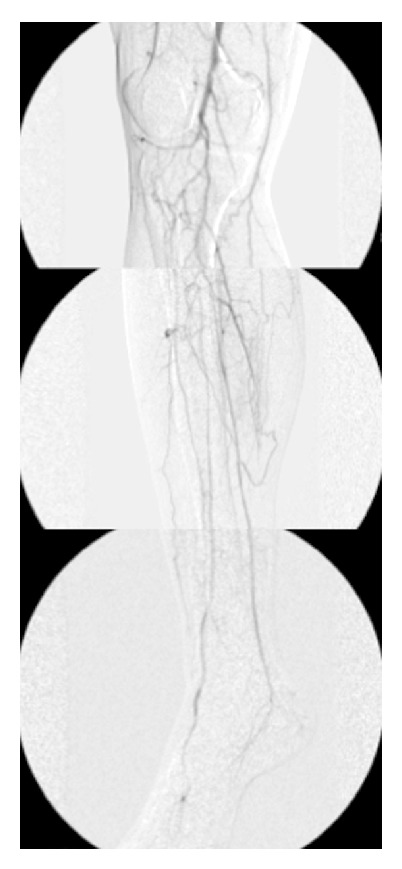
Case 2: preoperative arteriography showed that the popliteal artery was occluded, but there were no abrupt interruptions in the tibial or peroneal arteries.

*Case 2.* A 28-year-old male presented at another hospital with a three-year history of intermittent claudication in the left lower limb and coldness in the toes. He had no cardiovascular risk factors, such as diabetes mellitus, dyslipidemia, or a smoking habit. However, angiography showed occlusion of both popliteal arteries and widespread obstruction of the crural arteries, which led to an initial diagnosis of Buerger disease (Figure 3). The patient was then transferred to our hospital. According to the clinical examinations, the bilateral femoral, popliteal, and brachial arteries were palpable; however, the pedal pulses on the right side were absent. The ABI on the right side was 0.50, while that on the left side was within the normal range.

The laboratory investigations showed no abnormalities, including a hypercoagulable state or autoimmune or infectious diseases. However, computed tomography (CT) revealed compression of the right popliteal artery from the medial head of the gastrocnemius muscle as well as the left popliteal artery due to an accessory slip of the gastrocnemius muscle. The clinical findings did not fulfill the diagnostic criteria for Buerger disease, and the CT results showed that the structure of the bilateral popliteal fossa had caused the ischemia. Therefore, we corrected the diagnosis to popliteal artery entrapment syndrome (PAES) rather than Buerger disease and treated the patient surgically.

We subsequently performed right femoroposterior tibial bypass with autogenous vein and left resection of the accessory slip of the medial head of the gastrocnemius muscle. The patient's postoperative course was uneventful, and the claudication was relieved after the surgery.

## 3. Discussion

Buerger disease is primarily diagnosed based on clinical symptoms. We use Shionoya's criteria [6] to diagnose Buerger disease at our institution. These criteria include five clinical signs: a history of smoking; onset before 50 years of age; infrapopliteal arterial occlusive disease; upper limb involvement or phlebitis migrans; and the absence of atherosclerotic risk factors other than smoking. Imaging modalities should be used to identify the distribution of arterial involvement. Angiography is essential for making an accurate diagnosis of Buerger disease. Typical arteriographic findings, such as abrupt or tapering occlusion, corkscrew collaterals, and the absence of calcification, provide supporting evidence for a diagnosis of Buerger disease [7]. Biopsy and tissue sampling are rarely needed to confirm the diagnosis of Buerger disease; however, in rare cases with an unusual onset of symptoms, the histopathological findings are useful for making the definitive diagnosis [8].

FMD is a nonatherosclerotic, noninflammatory arterial disease that usually affects the small and medium arteries, followed by luminal narrowing and aneurysm formation [9]. The disease occurs in young patients with few cardiovascular risk factors, and the most distinctive clinical feature of FMD is the distribution of the affected arteries. Notably, the renal arteries (79.7%) and extracranial carotid artery (74.3%) are highly involved in cases of FMD [10]. However, in the current Case 1, the renal and carotid arteries were intact. The onset of FMD in the peripheral arteries, as noted in Case 1, is very rare [11]. FMD is divided histopathologically into five types according to the affected arterial layer: intimal fibroplasia, medial fibroplasia, perimedial fibroplasia, medial hyperplasia, and adventitial fibroplasia [12]. In Case 1, the arterial wall was thickened primarily in the media, and hyperplasia was seen in the adventitia. Therefore, the patient was diagnosed with the perimedial fibroplasia type of FMD. It is interesting that Case 1 was diagnosed as Buerger disease according to Shionoya's clinical criteria and then rediagnosed as FMD based on the histopathological findings. This suggests that FMD may have been present in previous cases of Buerger disease.

In Case 2, Buerger disease was suspected based on the arteriographic findings of diffuse infrapopliteal arterial occlusion. However, the patient had neither a smoking habit nor upper limb involvement. Therefore, we rediagnosed the case as non-Buerger disease, and the CT findings showed abnormal bilateral musculotendinous structures surrounding the popliteal fossa, which led to the correct diagnosis of PAES. The majority of PAES cases have been reported in young males, with over half of patients diagnosed before 30 years of age. Popliteal artery compression by abnormal slips causes intimal damage, thrombosis, and distal embolization [13], which leads to ischemic damage and eventual limb loss. Surgical management requires releasing extrinsic compression and restoring the arterial flow. Myotomy of the abnormal muscle and/or revascularization procedures are required. Sympathectomy is effective for Buerger disease; however, patients with PAES require release of the arterial compression, not sympathectomy, which is the only possible treatment for relieving the ischemic symptoms. Therefore, PAES should be promptly and accurately diagnosed so that it can be effectively treated in order to prevent the development of severe irreparable ischemic complications. Buerger disease is similar to PAES in the points of the young age of onset and male predominance. However, Buerger disease is strongly correlated with smoking, and the disease generally occurs in young smokers, with episodes of remission being correlated with smoking cessation and relapse being correlated with restarting smoking [14]. In cases such as Case 2, in which the patient did not have a history of smoking, the possibility of peripheral arterial diseases other than Buerger disease should be considered initially because Buerger disease is strongly associated with smoking.

In conclusion, Buerger disease is a clinical diagnosis that requires a compatible history, supportive physical findings, and the presence of diagnostic vascular abnormalities on imaging studies. Laboratory tests, including assessments of the autoimmune system and a hypercoagulable state, are used to exclude alternative diagnoses in patients with suspected Buerger disease. Distal small to medium artery involvement, segmental occlusion, and a “corkscrew” appearance of collaterals are typical angiographic findings in patients with Buerger disease. Diagnosing Buerger disease is not usually difficult in typical cases, although uniform criteria are lacking. Therefore, physicians should be aware that it is possible that other occlusive diseases, such as FMD and PAES, are to be misdiagnosed as Buerger disease in some cases.
